# The Herbst appliance and its modifications - prevalence and individuality

**DOI:** 10.1186/s13005-021-00266-2

**Published:** 2021-05-05

**Authors:** Mareike Karbach, Claudia Zöller, Georg Zöller, Heinrich Wehrbein, Christina Erbe

**Affiliations:** 1 Department of Orthodontics, University Medical Center of the Johannes Gutenberg-University, Augustusplatz 2, D-55131 Mainz, Germany; 2 Orthodontic Practice, Pirmasenser Straße 59, 67655 Kaiserslautern, Germany

**Keywords:** Herbst appliance, Modification, Class-II-malocclusion, Anchorage

## Abstract

**Objective:**

The aim of this study was to analyze the use of modified, cast splint Herbst appliances for the treatment of skeletal class II as an alternative to surgical bite correction over a period of five years.

**Materials and methods:**

The patient cases all originate from the patients of the Department of Orthodontics at the University Medical Center of the Johannes Gutenberg University Mainz, Germany and the orthodontic practice Dres. Zöller, Kaiserslautern, Germany. Inclusion criteria were orthodontic treatment with the Herbst appliance and its modifications. The type of modification, number and frequency of the different modifications were determined on the basis of patient files, X-ray documents, photos and models.

**Results:**

Of a total of 2881 new admissions over a period of five years, 1751 patients came from the Department of Orthodontics at the University Medical Center of the Johannes Gutenberg University Mainz and 1130 from the orthodontic practice in Kaiserslautern. A total of 336 patients were treated with a Herbst appliance during the period mentioned. 14 (13%) of the cases from the Herbst patient collective of the University Medical Center and 45 (19%) of the cases from the orthodontic practice were classified as modifications. The following modifications could be determined in descending order: University Medical Center Mainz: Herbst for anchorage during space closure (65%) > distalization (14%) ≥ bar construction as a space maintainer (14%) > Herbst applicance for anchoring for the adjustment of impacted teeth (7%); orthodontic practice Kaiserslautern: Herbst appliance with quadhelix in the maxilla (42%) > distalization (27%) > space closure (15%) > bar construction as a space maintainer (9%) > adjustment of impacted teeth (7%), multiple modifications occurred at 11%. The combination of quadhelix and Herbst appliance as well as multiple modifications have not yet been used in the University Medical Center Mainz. As an alternative to dysgnathia surgery, 23 adult patients (> 18 years) from the University Medical Center and 22 from the orthodontic practice were treated with a Herbst appliance.

**Conclusion:**

Nearly 12% of Herbst appliances are used in everyday orthodontic practice and almost 18% of these are used with modification(s). The high anchoring quality and force-effect geometry of the Herbst appliance is suitable for combining and treating various other treatment tasks in addition to the classical treatment task of class II therapy.

## Background

After the Herbst appliance was reintroduced in 1979 by Hans Pancherz, it is largely established in today’s orthodontics for Class II therapy [1]. Due to the frequent occurrence of the Class II anomaly, which is caused by mandibular retrognathia, the orthodontist is often confronted with this form of dysgnathia [2–6]. Nearly 1/3 of the European and US population have a Class II malocclusion [6]. There is a high demand for a therapy that is as compliance-independent as possible with a treatment time as predictable as possible [7–9]. Fixed devices for Class II correction provide an option for treating this form of dysgnathia after exceeding the pubertal growth peak and are also suitable in adulthood to avoid a surgical procedure for Class II correction [2, 10]. Efficiency for the dentist, integration into everyday practice as well as fast and good effectiveness led to the further development and variations of the fixed Class II mechanics [11, 12]. The current literature includes extensive investigations of the Herbst appliance on dentoskeletal effects, changes in the facial profile, muscle activity, anchoring problems (headgear effect and protrusion of the mandibular incisors), clinical complications, design of the appliance and comparisons between a surgical correction of class II and use of the Herbst appliance [8, 13–16].

In contrast, few studies are available on the variety of design options for the modification of the Herbst appliance and the resulting further treatment options [17]. This is particularly surprising in light of the fact that both soldered and cast splint variations are usually highly individualized designs. On the other hand, there are additive Class II mechanisms (e.g. SUS, MARA, Forsus) which can be attached to the multibracket appliance and are available as a ready-made set.

The Herbst appliance does not have to be used exclusively for the treatment of the class II jaw relationship. In combination and as a modification, it can be a multifunctional treatment tool and can be used, for example, instead of minipins as an anchoring element when closing spaces in the lower jaw [17]. Metzner et al. even reported a significantly faster space closure when using the Herbst appliance as an anchoring tool in combination with a lingual bracket system than space closure and TADs (temporary anchorage device) [17]. Modifications of the Herbst appliance faciliate the management of different treatment tasks and are non-invasive. This can, for example, avoid a surgical procedure if the indication is correct [13, 18–20].

The aim of this study was to analyze the use of modified, cast splint Herbst appliances for treatment and as an alternative to surgical interventions over a period of five years.

## Materials and methods

All examined patients are from the patient collective of a university medical center specialized in the Herbst appliance treatment as well as an independent practice. A cast splint Herbst appliance was used for all patients from the practice and clinic. The average age was 15.6 ± 5.61 years. 53.3% of the patients were female and 46.7% were male. The type of modification, number and frequency of the individual modifications were determined on the basis of the patient files, X-ray documents, photos and models. All anchor teeth in the upper and lower jaw had erupted at the time of insertion of the Herbst appliance. Successful insertion of the cast splint Herbst appliance as well as regular check-ups and final removal of the appliance were prerequisites for inclusion in this study. From these preselected patients with a cast splint Herbst appliance, all patients treated with a conventional cast splint Herbst appliance were eliminated. This means that in the upper jaw the first molars as well as the first and second premolars and in the lower jaw the canines (only in University Medical Center), first and second premolars as well as the first molars in the form of rigid splints are included in the appliance. The upper and lower jaw splints are connected by a telescopic mechanism and behind the teeth of the lower jaw, a cast splint lingual arch connects the two lower splints.

The patient group, which was treated with the conventional cast splint appliance for the correction of the skeletal class II, serves exclusively to determine the frequency of the integration of the Herbst appliance and to set it in relation to the modified appliances.

The inclusion criteria include modifications in the sense of a cast splint bar construction to maintain a space exclusively in the mandibular posterior region. Further criteria include distalization and mesialization mechanisms with the help of molar bands, molar tubes, buttons and partial arches, which were combined by soldering with the conventional cast splint herbst appliance. Also included were anchoring aids such as cantilevers and soldered-on tubes for adjusting displaced and impacted teeth and the combination with a quadhelix. Premature loosening and complications in terms of appliance fractures and recementing were not considered in this study.

## Results

**Fig. 1:**
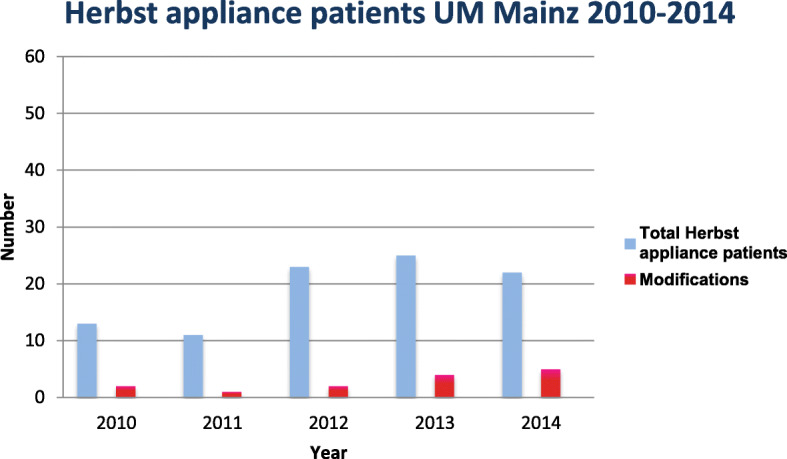
Total number of Herbst appliance patients of the Department of Orthodontics at the University Medical Center of the Johannes Gutenberg-University Mainz, Germany, divided into modification

Out of a total of 2881 new admissions in the specified study period, 1751 patients came from the Department of Orthodontics at the University Medical Center of the Johannes Gutenberg–University of Mainz and 1130 from the orthodontic practice Kaiserslautern. A total of 336 patients, corresponding to 11.7%, were treated with a Herbst appliance. Patients from both facilities were treated before, after or at the growth peak. From the Herbst patient collective of the University Medical Center Mainz 14, i.e. 13% of the cases and from orthodontic practice Kaiserslautern 45, i.e. nearly 19% of the cases were classified as a modification. Overall, almost 18% of Herbst patients at both institutions were classified as modifications (*n* = 59). The following modifications were identified in descending order:

At the University Medical Center, 65% (*n* = 9) of the modified Herbst appliances were used to close a space in the posterior region. In 14% (*n* = 2), it was used as a distalization mechanism in the posterior region and provided with a bar construction as a space maintainer. In 7% of the cases, impacted and displaced teeth could be adjusted after surgical exposure with the help of the Herbst appliance (*n* = 1) (Figs. 1, 3 and 4).

**Fig. 2:**
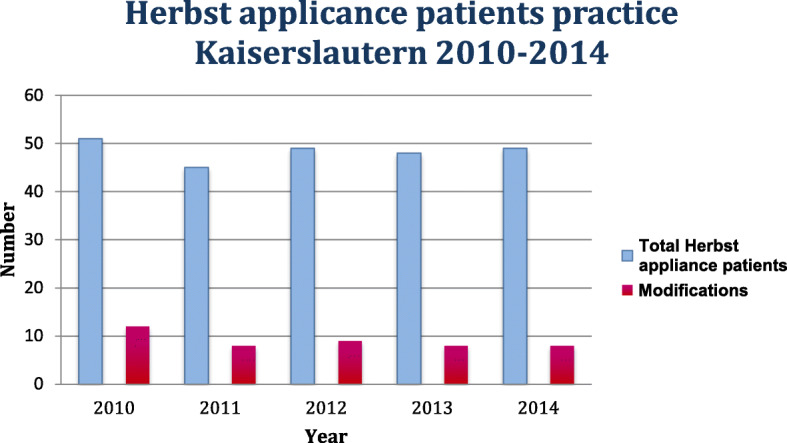
Number of Herbst appliance patients of the orthodontic practice Dres. Zöller, Kaiserslautern, Germany, total and divided into modification

**Fig. 3:**
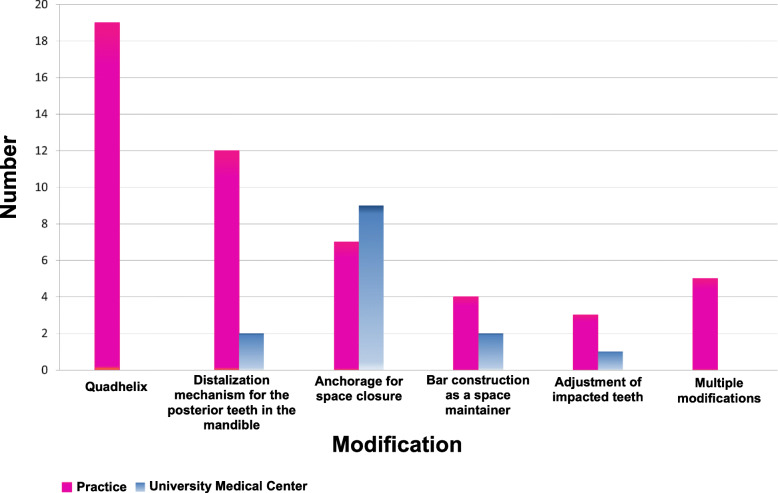
Classification according to type of modification; Department of Orthodontics at the University Medical Center of the Johannes Gutenberg-University Mainz, Germany (blue), and orthodontic practice Dres. Zöller, Kaiserslautern, Germany (pink)

**Fig. 4:**
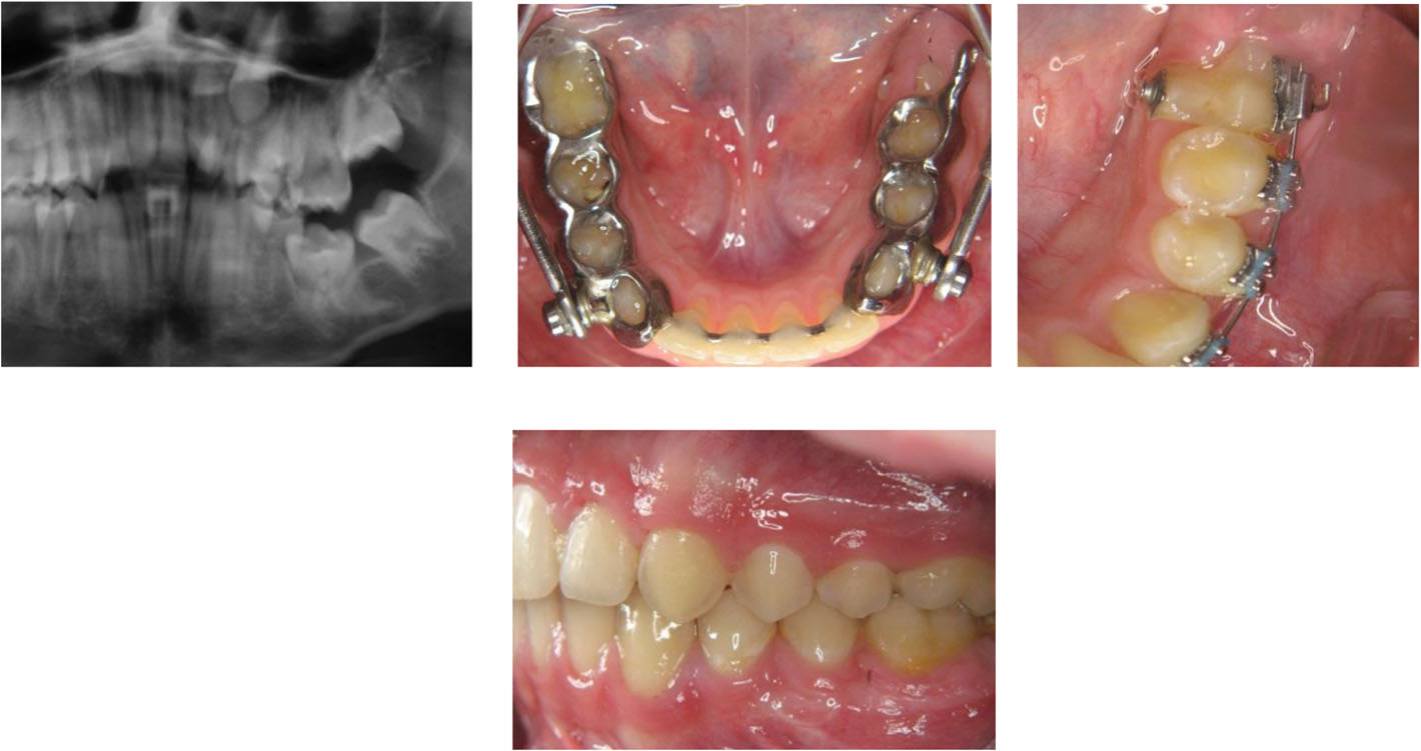
Patient example: Impacted and displaced tooth 36 in a 12-year-old patient; **a** Partial section of the pretherapeutic OPTG, **b** Herbst appliance in situ with cantilever Regio 36 for adjustment of tooth 36 (**c**) After Herbst treatment: Partial section with multibracket appliance in the lower left quadrant (**d**) Lateral photo at end of treatment with aligned tooth 36

In the specialist practice, 42% (*n* = 19) of the Herbst appliances were used in combination with a quadhelix to correct a transverse deficit in the maxilla > 4 mm and simultaneously to correct class II malocclusion. It was used in 27% (*n* = 12) of the cases as a distalization mechanism for the posterior teeth in the mandible. The Herbst appliance was used in 15% (*n* = 7) of cases as an anchoring tool for space closure in the posterior region of the mandible. Also, bar constructions in the posterior region in the sense of a space maintainer were used in 9% (*n* = 4) of the cases and the use of the Herbst appliance for the adjustment of impacted teeth occurred in 7% (*n* = 3) of the cases. Multiple modifications in one appliance occurred at 11% (*n* = 5) (Figs. 2, 3 and 4). The combination of a quadhelix and Herbst appliance as well as multiple modifications were not applied in the University Medical Center during this period. As an alternative to orthognathic surgery, 23 adult patients (> 18 years) from the University Medical Center and 22 from the orthodontic practice were treated with a Herbst appliance.

## Discussion

The use of the Herbst appliance as an anchoring tool and modification for various treatment tasks is hardly established in orthodontics and only rarely described in the literature [17, 18].

The cast splint Herbst appliance belongs to the rigid, bimaxillary Class II mechanisms, which allow the mouth to be opened by means of a telescopic mechanism and the lower jaw to be held in the desired target position [1]. In this study, only cast splint Herbst appliances were used to allow modifications to the appliance itself. Due to its support on several anchor teeth in the upper and lower jaw, it can be used as a multifunctional anchoring tool. The Herbst appliance can also be used as a passive anchoring tool if a class II malocclusion is not present.

If additional correction of the class II jaw relationship is desired, the Herbst appliance is the most effective treatment device [7, 11, 16]. The ideal time for insertion of the Herbst appliance is indicated after the pubertal growth peak until the end of pubertal growth [10]. In this study, the mean age was 15.6 years (σ = 5.6). This insertion period is supported by studies on treatment with the Herbst appliance also in early adulthood or later adolescence and produces comparable success for Class II correction as immediately after the pubertal growth peak [10, 21].

The gender distribution also corresponds to previous studies on Class II treatment with the Herbst appliance [11, 15].

**Fig. 5:**
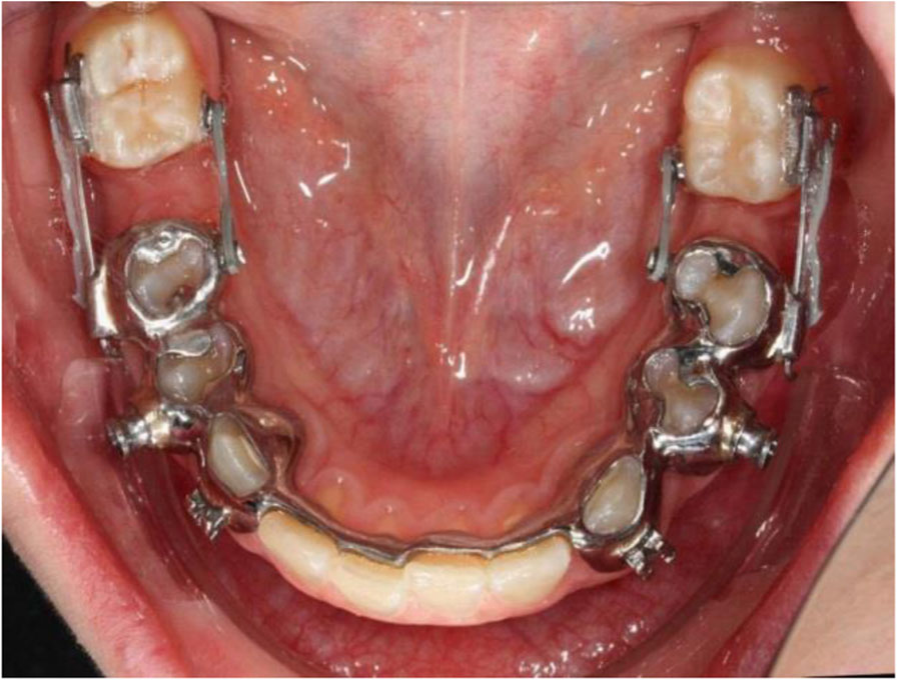
Patient example: Space closing on both sides in the lower jaw region 36 and 46 with the Herbst appliance as anchoring unit in a case of aplasia of teeth 35 and 45 (for better visualization the telescopic bars were removed before the photo was taken)

Due to the multifunctionality of this cast splint Herbst appliance, which could be shown in this retrospective study, the patient’s wish for as non-invasive a procedure as possible can be fulfilled. Furthermore, fixed dentures and implants can be avoided, for example, in the case of aplasia of teeth with previously performed orthodontic space closure (Fig. 5). In addition, compensation extractions can also be avoided due to a lack of experience on the part of practitioners with regard to the possibility of therapy with the Herbst appliance to close the space if the indication is correct and the ideal function and aesthetics are nevertheless achieved [22, 23].

Springy, bimaxillary Class II mechanics such as the Forsus spring, Jasper jumper and Sabbagh Universal Spring (SUS and rigid Class II mechanics such as the mandibular anterior repositioning appliance MARA leave little room for modifications to the appliance itself and thus the solution of multifunctional treatment tasks due to its design. Here, the delicate design and the resulting increased wearing comfort play a greater role for the patient and can be used almost exclusively for sagittal mandibular correction in the anterior direction if there is a transverse congruence of both jaws [12]. Zimmer et al. describe in their study the use of Jasper Jumper for uni- as well as bilateral space closure in the mandible after anchorage loss in the anterior mandiblewith compression-tension mechanics and class II elastic bands [23]. These push-pull mechanics and class II rubber bands require a high level of patient cooperation and compliance. The use of the Herbst appliance as anchorage for space closure in the molar region of the lower jaw in combination with a Class II treatment was 65% prevalent in the University Medical Center and 15% in the orthodontic practice during the study period (Fig. 5). In the literature, the occurrence of aplasia of teeth is stated as approx. 6.7%. This affects 41% of the second lower premolars and 13% of the first lower premolars [24]. Anchoring problems to close the space can thus also be solved in combination with a class II malocclusion. Studies on the use of the Herbst appliance for mesialization of molars in the mandible in combination with a lingual bracket system can be found [17]. Here, Metzner et al. were even able to determine significantly faster mesialization of mandibular molars with the Herbst appliance as anchoring tool and a lingual bracket system than with TADs as an anchoring tool [17]. If the indication is correct before the start of therapy, the patient can be spared an invasive, painful surgical procedure and additional costs [25]. On the other hand, there are numerous studies that use TADs as an anchoring tool for the mesialization of lower jaw molars and premolars to close spaces, for example [19, 25].

**Fig. 6:**
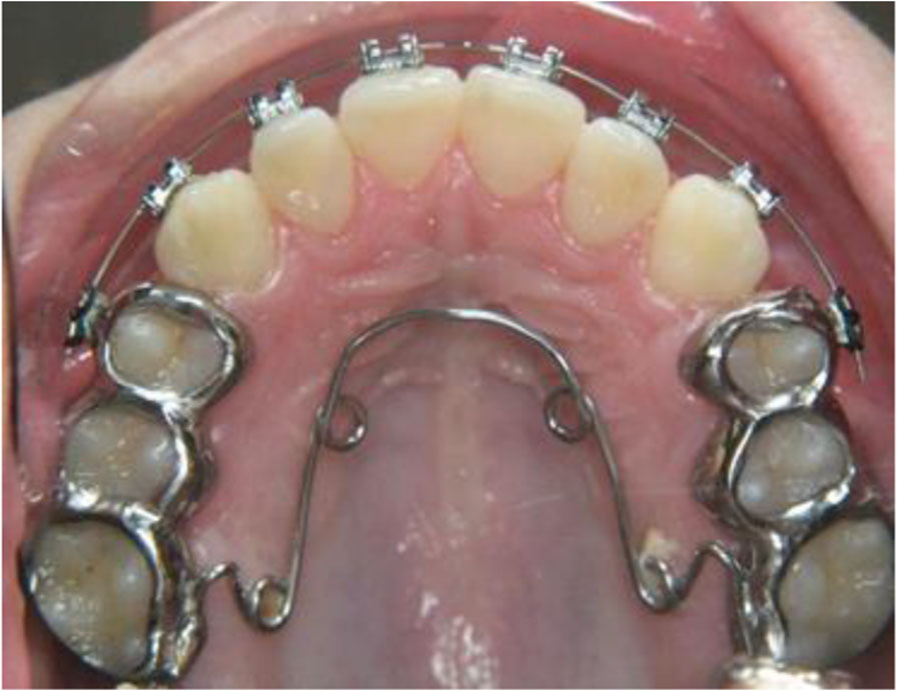
Patient example: Herbst appliance in combination with a quadhelix before removal of the Herbst appliance; a transverse deficit > 4 mm and a ½ cusp Class II jaw relationship before start of treatment

The use of the Herbst appliance in combination with a quadhelix in the maxilla was only used in the specialist practice during the study period (Fig. 6). A transversal problem in the upper jaw with a deficit > 4 mm is treated in the Department of Orthodontics at the University Medical Center of the Johannes Gutenberg-University Mainz with rapid maxiullary expansion (RME) before the insertion of the Herbst appliance. In their book “The Herbst Appliance”, Pancherz and Ruf describe both possibilities, i.e. the Herbst appliance in combination with a quadhelix and an RME appliance before the Herbst appliance. In case of a large transversal deficit, treatment with an RME appliance should be performed before sagittal correction [18].

Due to the frequently expressed desire for a therapy concept that is non-invasive, preserves teeth and avoids prosthodontics, orthodontists are often confronted with the task of finding therapy solutions. This study aims to propose solutions for a multifunctional management of treatment tasks and to thus expand the orthodontic therapeutic spectrum.

The present retrospective study was prepared to investigate and present the frequency, prevalence and possible individuality of the Herbst appliance. The therapy result was not evaluated. Therefore, no statement can be made about the effectiveness of the different Herbst appliance modifications. This remains the subject of further investigations.

## Conclusion

12% of Herbst appliances are used in everyday orthodontic practice, and almost 18% of these are modified. The high degree of anchorage of the Herbst appliance enables multifunctional anchorage for the simultaneous therapy of various other treatment tasks as well as cost- and time-efficiency, in addition to the original class II correction. It appears logical to adapt the Herbst appliance to the treatment tasks in the form of individual modifications; the time of treatment – generally post-puberty – should be reconsidered.

The multiple modification possibilities have been under-investigated thus far. This suggests that so far there has been little use of the Herbst appliance for the “classic” indication of dentoalveolar and skeletal Class II malocclusion therapy.

For the planned closure of the spaces in the posterior lower jaw area, TADs have so far been used as anchorage rather than fixed bimaxillary devices such as the Herbst appliance.

## Data Availability

The data used for analysis has been referenced in the text or tables of the paper.

## Abbreviations

TAD: Temporary anchorage device
SUS: Sabbagh universal spring
MARA: Mandibular anterior repositioning appliance
RME: Rapid maxillary expansion
